# The RHD Action Small Grants Programme: Small Investment, Big Return!

**DOI:** 10.5334/gh.996

**Published:** 2021-04-27

**Authors:** Leila Abdullahi, Cleo Albertus, Susan Perkins, Kate Ralston, Alastair White, Jeremiah Mwangi, Liesl J. Zühlke

**Affiliations:** 1 African Institute for Development Policy (AFIDEP). Nairobi, KE; 2 Reach, Cape Town, ZA; 3 World Heart Federation, Geneva, CH; 4 Reach, Geneva, CH; 5 Division of Pediatric Cardiology, Department of Pediatrics, Red Cross War Memorial Children’s Hospital, University of Cape Town, Cape Town, ZA; 6 Division of Cardiology, Department of Medicine, Groote Schuur Hospital, University of Cape Town, Cape Town, ZA

**Keywords:** rheumatic heart disease, small grants, world health organisation resolution

## Abstract

Rheumatic Heart Disease (RHD) remains endemic in low- and middle-income countries (LMICs) despite its virtual elimination in high-income countries. RHD Action was launched to amplify global efforts to control RHD in 2015 by World Heart Federation and Reach, with demonstration projects in Uganda and Tanzania, and support from Medtronic Foundation. The Small Grants Programme focuses on three domains: People and Communities, Medicines and Technologies, and Systems and Services. It is designed to support patient and community groups in promoting awareness, advocacy, and to build health workers’ capacity to prevent and treat RHD in LMICs. Our study evaluates the impact and effectiveness of the RHD Action Small Grants Programme.

**Methods:** We conducted a mixed method study that involved both quantitative and qualitative surveys, through phone interviews and online surveys amongst the grant beneficiaries, to assess the impact and effectiveness of the small grant programme. An invitation to complete an online survey, using a Google Forms format, was issued to Small Grant Project Directors and Co-Directors that received funding for projects between 2017 and 2019. The online survey requested basic project information using tick boxes, Likert scales, and short answer open-ended questions about successes and challenges faced by recipients. The questionnaire also addressed recipients’ experience with the RHD Action Small Grants process – applying for the grant, nature and quality of support received to carry out project, the reporting process, and any media coverage provided. For the phone interviews, responses to the short-answer questions were used as the basis for follow up phone interviews. The discussions were recorded, transcribed and thematically analysed for new and recurring themes emerging from the in-depth discussions.

Initiated in 2017, RHD Action has funded 21 proposals from a pool of 60 submissions. Recipient countries include Zambia, Uganda (2), Namibia, Kenya, Malawi (2), Egypt, Ethiopia, Nigeria (3), Rwanda (2), Mozambique, and Cameroon (2) as well as Fiji (2), the Philippines and Nepal. Five recipients were funded in 2017, eight in 2018 and eight in 2019. Project directors are primarily junior doctors and project managers supervised by senior mentors. In most cases, this is their first funding award. These projects have demonstrated tangible impact and have provided content for first manuscript and abstract submissions and presentations at professional conferences. Grant reports are presented as website stories showcasing the achievements of small local efforts with meaningful impact.

For RHD Action, there is large return on a modest monetary investment resulting in a very visible, viable global RHD networking platform for enthusiastic community and provider activists.

## Background

Rheumatic Heart Disease (RHD) remains endemic in low- and middle-income countries (LMICs) despite its virtual elimination in high-income countries. RHD Action was launched to amplify global efforts to control RHD in 2015 by the World Heart Federation and Reach, with demonstration projects in Uganda and Tanzania, and support from Medtronic Foundation. The Small Grants Programme focuses on three domains: People and Communities, Medicines and Technologies, and Systems and Services. It is designed to support patient and community groups in promoting awareness, advocacy, and to build health workers’ capacity to prevent and treat RHD in LMICs.

The RHD Action Small Grants Programme commenced in 2017 as a collaboration between Reach and the World Heart Federation. The Programme was conceived as a mechanism for sponsoring and supporting small, grassroots, RHD-related activities in LMICs where RHD is endemic. Individual awards were limited to $2000 for the first round and increased to $2500 thereafter. Three requests for proposals were issued between 2017 and 2018 resulting in a total of 13 awards. Two requests for proposals have been issued in 2019, with an expectation of funding eight additional proposals. As the five-year RHD Action award approaches its end in April 2020, the funder, Medtronic Philanthropy, has requested a recap and assessment of the impact of the Small Grants Programme.

The purpose of this assessment is to evaluate the impact and effectiveness of the RHD Action Small Grants Programme by asking individuals whose projects were successfully funded to provide feedback on their experiences – positive and negative – with the Programme.

### Objective

To evaluate the impact and effectiveness of the RHD Action Small Grants Programme.

## Methods

### Award selection process

Requests for proposals were issued through RHD Action, PASCAR and WHF email distribution lists and cross-promoted on websites. A review panel with representation from Reach, WHF and demonstration projects used structured criteria for scoring based on clear, feasible, measurable objectives. Preference was given to proposals with dissemination plans using local publicity and social platforms, and for garnering support from local MOH officials. Final selections were approved by Medtronic Foundation. Funding increased from $2,000 to $2,500 after the first round.

### Study evaluation

This was a mixed method study that involved both quantitative and qualitative surveys through the following channels;

1. Online survey

An invitation to complete an online survey, using a Google Forms format, was issued in Dec 2019 to Small Grant Project Directors and Co-Directors for projects funded between 2017 and 2019. The online survey requested basic project information using tick boxes, Likert scales, and short answer open-ended questions about successes and challenges faced by recipients.

The questionnaire addressed recipients’ experiences with the RHD Action Small Grants process – applying for the grant, nature and quality of support received to carry out the project, the reporting process, and any media coverage generated.

Respondents were asked to indicate if they would agree to be contacted for a phone interview and if so, to provide best contact details. Completing the online survey required 15-25 minutes for completion. Recipients were given a four-week response window that occurred over the holiday period. Three follow-up attempts were made to any non-responders. The respondent identity was concealed, and a random anonymous number was used for identification.

2. Phone interviews

Responses to the short-answer questions were used as the basis for follow-up phone interviews. These discussions were recorded, transcribed and thematically analyzed for new and recurring themes emerging from the in-depth discussions. The phone interview took about 30 minutes to be completed.

### Ethics approval

The activity was framed as an “Impact Assessment/Evaluation” activity for a single programme. Therefore, ethics approval is not required.

## Results

### Characteristics of the grant recipient

The RHD Action Small Grants Programme was initiated in 2017, 21 proposals have been funded from a pool of over 60 submissions. Recipient countries include Zambia, Uganda (2), Namibia, Kenya, Malawi (2), Egypt, Ethiopia, Nigeria (3), Rwanda (2), Mozambique, and Cameroon (2) as well as Fiji (2), the Philippines and Nepal. Five recipients were funded in 2017, eight in 2018 and eight in 2019. Project directors are primarily junior doctors and project managers supervised by senior mentors. For most of the beneficiaries, The RHD Action Small Grants Programme is their first funding award.

Among the funding beneficiaries, 14 responded to the qualitative questionnaires and 16 participants responded to the quantitative questionnaire. Table 1 shows a summary of the grant beneficiaries who responded to the quantitative survey aggregated based on the year of the grant. For both quantitative survey and qualitative interviews there was a 76% response rate with most of the respondents being grant recipients from 2018 and 2019, Figure 1 shows the distribution of respondents below.

**Figure 1:**
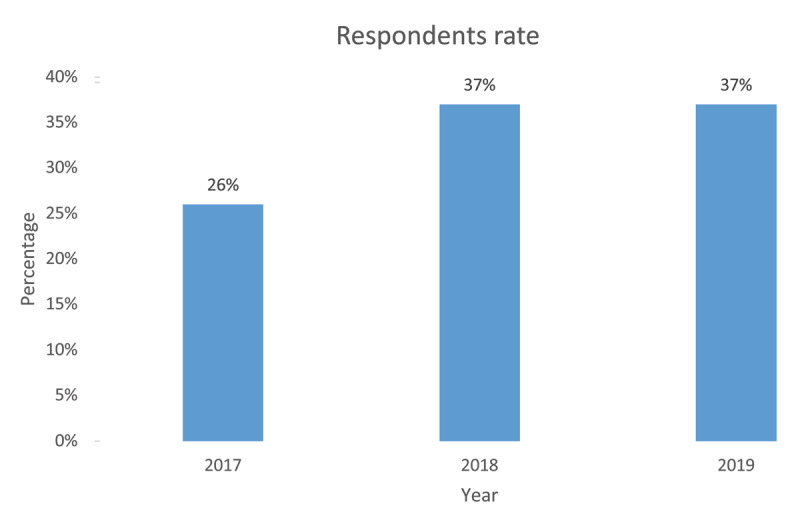
Respondent rate among grant recipients.

**Table 1:** Characteristics of the grant recipient and beneficiaries.

	Year	Country	Small Grant Recipient project	Beneficiaries	Sample size

**1**	2018	Nigeria	Kick RHD Out of Plateau State: Reaching out to Berom- and Hausa-speaking communities	PLWRHD	140
**2**	2018	Cameroon	Initiating a Rheumatic Heart Disease Clinic in Yaoundé, Cameroon	PLWRHD	45
				Doctors, Nurses, Allied Health Professionals	6
**3**	2018	Mozambique	Reproductive Health Services and Cardiovascular Health: The RESCUE RHD Project	Doctors, Nurses, Allied Health Professionals	80
				PLWRHD	15
**4**	2018	Nepal	Knowledge, Attitude and Practice (KAP) of Rheumatic Heart Disease among Health Care Providers in Eastern Nepal: Measuring the Impact of an Educational Intervention	Doctors, Nurses, Allied Health Professionals	123
**5**	2018	Nigeria	Advocacy and Capacity building of community health workers in Osun State, Nigeria	Front-Line Health workers	320
**6**	2018	Rwanda	Improving Health Education Among Post-Operative RHD Patients	PLWRHD	160
				Doctors, Nurses, Allied Health Professionals	2
**7**	2017	Kenya	Hearts to Hearts: A Rheumatic Heart Disease (RHD) Awareness Campaign.	School Based: Children, Teachers, Parents	10670
**8**	2017	Fiji	Empowering and Supporting Young People Living with RHD (PLWRHD) Activities: Mosquito Island	PLWRHD	113
**9**	2017	Zambia	Championing sexual and reproductive health among female adolescents and women living with RHD	PLWRHD	28
**10**	2017	Malawi	Acute Rheumatic Fever and Rheumatic Heart Disease Workshops in Lilongwe, Malawi	Doctors, Nurses, Allied Health Professionals	65
**11**	2018	Philippines	Creation of Standardized RHD Educational Materials to Support and Promote the National Implementation of Free Secondary Prophylaxis in the Republic of the Philippines	Doctors, Nurses, Allied Health Professionals	90
**12**	2018	Uganda	Rheumatic Heart Disease Education and Awareness in Nakivale Refugee Settlement	School Based: Children, Teachers, Parents	1428
				Doctors, Nurses, Allied Health Professionals	48
**13**	2017	Namibia	Namibian RHD Ambassadors Project; Attendance at 2 public events (estimate)	PLWRHD	90
**14**	2019	Egypt	Raising awareness about RHD in Alexandria	PLWRHD and community	2030
**15**	2019	Ethiopia	Implementation of educational training and screening of school-age children for rheumatic heart disease in North Ethiopia	PLWRHD and community	3700
**16**	2019	Cameroon	RHD Patient Empowerment for Community Awareness	PLWRHD and community	2550
**17**	2019	Malawi	Rheumatic Heart Disease Awareness Campaign	PLWRHD and community	357
**18**	2019/2020	Fiji	Investing and Empowering Community Health Workers through Motivational Interview training	Front-Line Health Workers	81
**19**	2019/2020	Rwanda	Voluntary based RHD Action Project (VOBRA-Project)	Front-Line Health Workers and community	4628
**20**	2019/2020	Uganda	Decentralization of BPG Best Practices, building the tools for a sustainable and scalable program.	Nurses and Front-Line Health Workers	45
**21**	2019/2020	Nigeria	Building community health workers’ capacity – kick RHD out of plateau state phase 2	Front-Line Health Workers	75

** PLWRHD- people living with Rheumatic Heart Disease.

### Grant recipient project completion

Of the recipients who responded, half of them completed the project and the other half are in progress. This is due to majority of recipients being awarded grants in 2019, with funds disbursed in 2020 (Table 1).

### Grant application process and award giving

**Figure 2:**
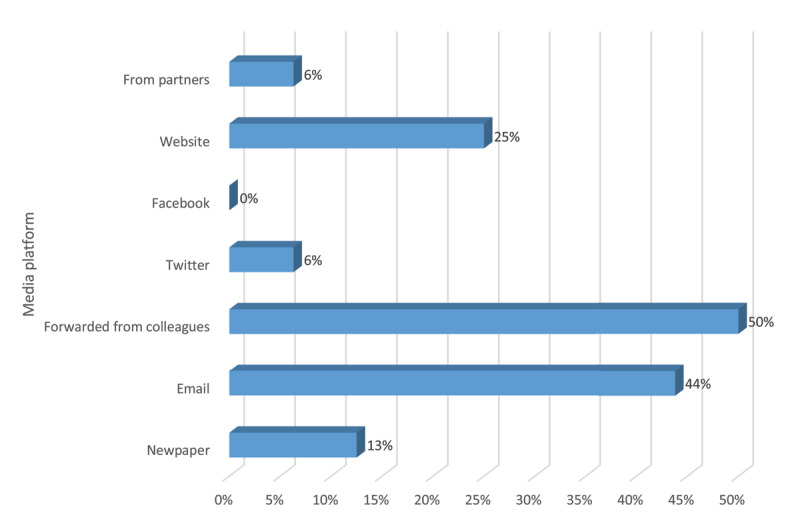
Digital platforms that recipients received the application message.

The grant application was shared through RHD Actions digital channels and cross promoted by partner organizations. Most recipients received the opportunity via emails and the website (Figure 2).

Overall, on a Likert scale of 1–5 (1 being not helpful and 5 being very helpful) all the respondents explained that they found the RFP and Guidelines very helpful for the application process. The qualitative interviews further revealed that the application process was straight forward.

‘The application was extremely straight forward and easy. The guideline was helpful and made the application process easy’.

In addition, on a likelihood scale of 1–5 (1 being not efficient and 5 being very efficient) all the respondents found the timeline between application to the awarding of the grant to be efficient (50%) and very efficient (50%) with quick turnaround.

Overall, the support provided by RHD Action to carry out the project was very responsive and helpful with all the respondents reporting a helpful and responsive support (25% helpful/responsive and 68% very helpful/responsive). In addition, all the respondents confirmed that the reporting process required by RHD Action was doable and very reasonable.

Over and above, one of the beneficiaries responded that the grant has gone way beyond to benefit the recipients.

And actually, the money is a lot of money. It doesn’t seem like a lot when you look at in numbers, but how much we were able to do with it has been impressive (Respondent 13).

And we still are using a lot of that money for our world heart day, printing T-Shirts that type of thing and whenever someone has a bigger idea, it’s also easy because of the initially boost we had, it’s easier to get other sponsors to assist us on something (Respondent 6).

### Impact on Healthcare Workers

**a) Impact of grants among various stakeholders:**

**Figure 3:**
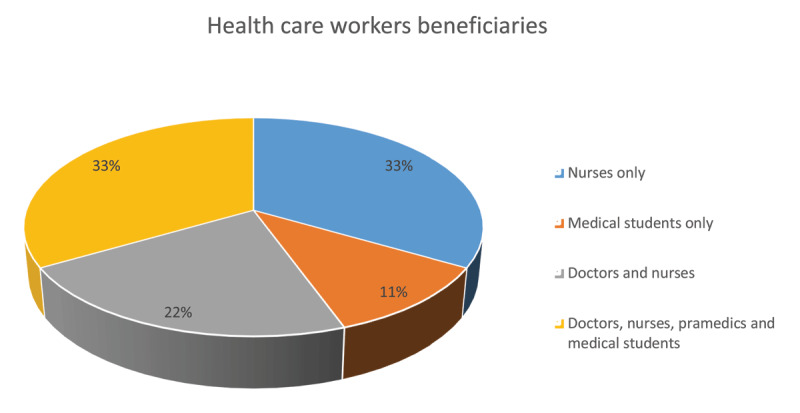
Platform to enhance health care workers awareness and knowledge.

Training was one of the platforms to enhance health care worker’s awareness and knowledge. Most of the trainings were conducted among health care workers at different career levels as shown in Figure 3 below.

**Figure 4:**
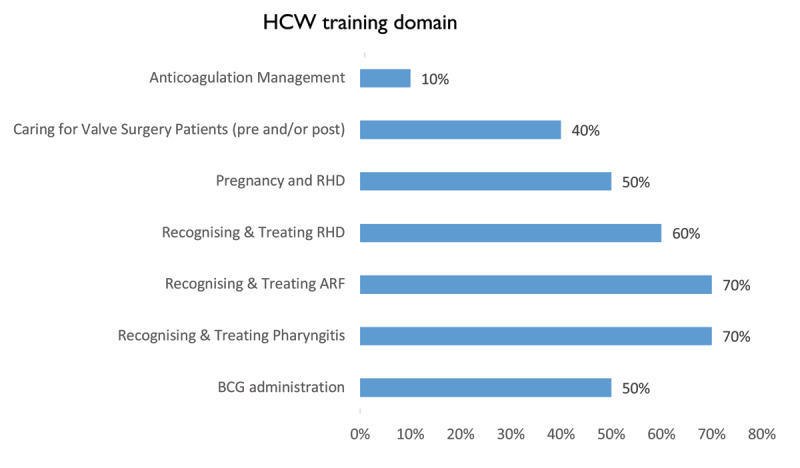
Health care worker RHD training domains.

The health care worker training explored all domains of RHD but mainly emphasized how to recognize and treat the condition (Figure 4).

The quantitative findings were investigated further using a qualitative survey where the health care workers explained the area of training that they benefited from.

previously on diagnostic skills on RHD, we didn’t know how to diagnose it, but with this project it has taken us very far. Currently, I am able to know if it is RHD or Rheumatic fever and compare. So, it has helped the diagnosis skills (Respondent 9).

when I got the RHD grant, facility X was struggling, most of the nurses were refusing to give penicillin because they had some bad outcomes as a result of the grant we were able to provide some pretty intense education and workshops to hospital staff to alleviate some of that fear. The health care workers after the workshops were more comfortable giving penicillin and improved their practice and care for kids with RHD (Respondent 10).

**b) Impact on the participant/patient and community**

Through education and awareness, the grant opportunity had a significant impact on the community. One of the respondents explained the impact of education on both patient (children) and parents:

it involved a lot of educating of not only the children who were the patients, but their parents as well, so they knew exactly what was happening and they knew what RHD, so they knew what they were dealing with? (Respondent 9).

The training and awareness targeted both health care facilities and non-health care facilities i.e. schools. Teachers were also involved to relay information to the community on management of RHD. The health care workers always engaged the parents when the children were involved.

We had an awareness and training campaign where we had targeted school-going children; some parents and teachers. The teachers were used as patrons for the children as a youth plan was formed, to assist to relay the information to the community (Respondent 9).

**Figure 5:**
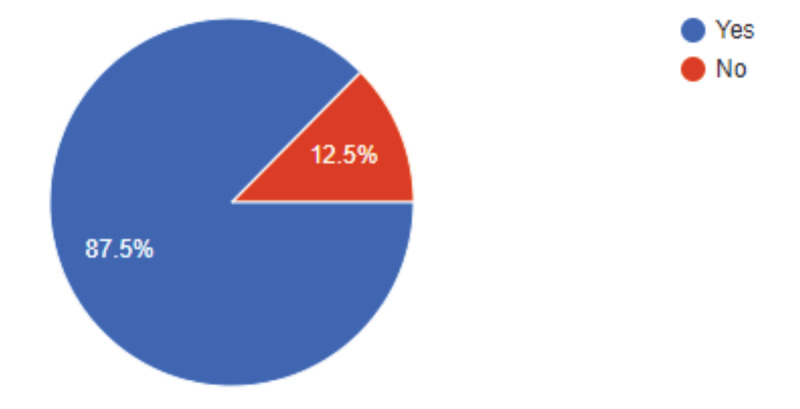
Proportion of beneficiary projects using digital media.

RHD Action provided digital media coverage of the work being done by the grantees (Figure 5). Among the grant recipients, 88% confirmed that they promoted their project using digital media and 69% using non-digital platform like posters/flyers. Facebook and twitter were the digital media most used among the beneficiaries.

**c) Impact on the organization**

The qualitative interviews discussed how the recipient felt the grant impacted their organization. Many recipients spoke in terms of their organization’s ability to carry out their work and sustain their mission. This was framed in terms of how patient and community awareness allowed them to be much more effective. The grant opportunity enhanced adherence and completion of treatment as advised by the doctor. During the patient visit, the organization created and enhanced awareness through education to the patient on the management of RHD.

So, now they are able to know quickly if someone was to have a sore throat. The community were educated on the importance of following the prescription and taking medication according to the doctor’s advice (Respondent 9).

The grant enabled us to help engage more with the community and stakeholders, so as a result staff and volunteers now have much more experience than we had before in community engagement (Respondent 3).

It was evident that before the grant the patients were non-adherent to treatment and follow up.

Many would take medication for five days and not finish the medication. So, the emphasis was taking the medication according to the doctor’s advice (Respondent 7).

The grant also helped to increase the feasibility of the organization and build more collaborations.

The grant has increased the feasibility on the organization. Both to the community and the healthcare workers on the facility (Respondent 8).

…. we could get more collaboration with the hospitals following the RHD grant, we actually trained more people and created more awareness, so I believe it was a bonus to move forward the RHD initiatives (Respondent 11).

The RHD grant did a very amazing impact on my organization, as it spread the impact of hours in the field and we just tried to get connected to other stakeholders all around the county. Therefore, the grant assisted us to get connected to other stakeholders who are interested in the field (Respondent 6).

**d) Impact on project director**

**Figure 6:**
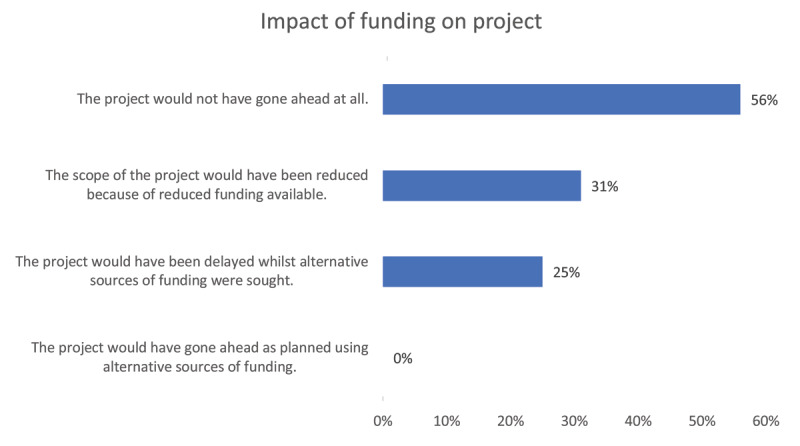
Relevance of the grant.

Most of the respondents expressed the relevance of the grant to themselves and the beneficiaries generally. Most of the project directors mentioned that the project would not have gone ahead if the grant was not given (Figure 6).

The qualitative survey alluded to the quantitative findings where the directors expressed that their ‘dream had come true’ through the grant opportunity. One respondent expressed that the opportunity of having grant has had such a huge impact on the RHD clinic.

There is a lot more awareness from the patients. There is a lot more patient support, the nurses, the healthcare professionals that work with our patients and even their family members because of the ambassador program and trying to get people involved in things (Respondent 14).

The directors of the project were motivated to work with the grant recipients/health care workers to make the best opportunity out of the programme. In one of the countries the project is working on empowering the patients and giving them a voice and their own obstacles.

Right now, they are working on making film, because we always said we would make a film, but because of the distances between us and the different patients due to COVID-19, and I mean I was quite surprised by, everyone wants to be in it. But you can’t have all six hundred people in a film (Respondent 14).

It is important to note that the grant boosted skills and confidence in many of the project directors. For some recipients it was the first grant they had received.

But for me personally, how can I say? the grant was like, a boost, so, a powerful way to encourage me (Respondent 1).

After receiving the grant, I think it opened up an opportunity to reach more people and I was able to meet people in the community who were at a higher level than I was. Hence, it opened connections and I was also able to be invited to conferences speak about it, meet more people and get more ideas. Generally, it broadened my network and I was able to learn more about the disease and how better I could help people (Respondent 4).

my care towards the patients also improved. I became more conscious and more, you know sensitive, looking out for people. Also, my professional profile improved in terms of the communication between other hospitals (Respondent 8).

**Figure 7:**
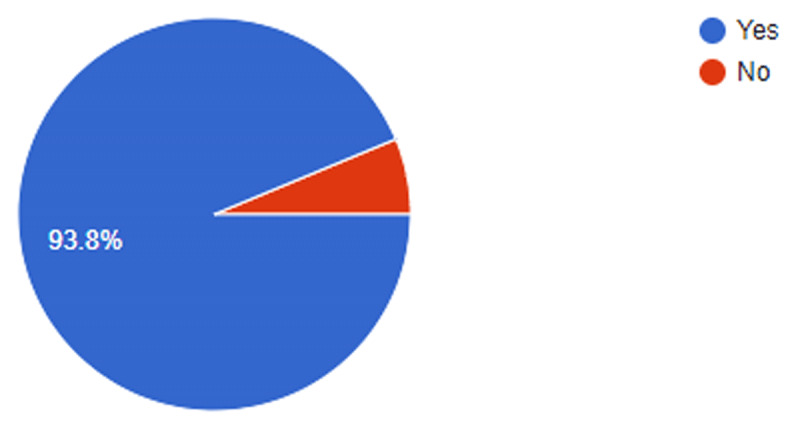
Proportion of directors working with other partners.

Collaboration was one of the keys the directors of the projects used to ensure project continuity even after the lapse of the grant. A majority (93%) of the directors worked in partnership with various stakeholders, that is, organizations/institutions/government officials in the delivery of their project (Figure 7).

### Communication platforms

**Figure 8:**
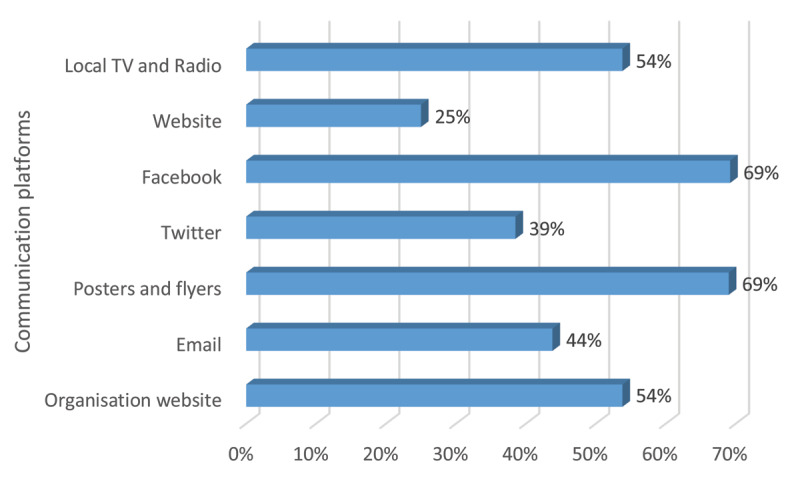
Common communication platforms.

Among the communication platforms, face book was one of the most used digital media among the beneficiaries as well as physical posters/fliers (Figure 8).

The grant recipients were creative to create a communication platform where the patients and the health care providers engaged to have a healthy discussion.

The grant has led to openness where it facilitated us having groups and community being more openness. The organization have created WhatsApp group where we have both patient and health care staff, I have actually never had that before, where I am on a WhatsApp group with my patients and I’ve said before you know, it’s not a medical group. It’s just a WhatsApp group, there is no ward, there is no penicillin, there’s no this, people can know, and we can all help each other at the hospital (Respondent 14).

### Government Engagement

A key criterion for selecting a grant was that the applications demonstrated how the grantee would engage the government in their work. Despite well thought out plans, grant recipients faced various challenges engaging their government.

The challenge is to bring the entire health sector and educational sector together, because I think if we can reach the entire health sector and educational sector, we can bring them together and reach the entire community (Respondent 12).

The challenges actually really were getting administrative approval, but actually we got some support from the military district in country X, they facilitated everything for us to access the region and to organize with the school because actually this time was exams and children were engaged in their exams (Respondent 2).

Yes, there were some challenges well some were political and in that we were having a national election at the time. So, we didn’t engage with one of the communities we had originally planned to do, because it was elections and it became quite intense and then eventually, we could not engage with that community. Yes, so political challenges (Respondent 3).

### Case studies on experiences on relevance of the grant to the recipient.

**Case extract 1: Kick RHD Out of Plateau State: Reaching out to Berom and Hausa speaking communities**

Between August and September last year (2018), Prof Bode-Thomas, Mr Santos Ayuba Larab and their team conducted five focus group discussions (FGDs) with targeted audiences from Berom, Hausa and English-speaking communities. There were over 140 participants representing families and patients who were awaiting heart valve surgery, post-operative valve surgery patients and their families, and general community audiences about recognising, treating and preventing RHD. Prof Bode-Thomas commented: “The FGDs were very well received. Community participants had many misconceptions about sore throats and no knowledge about its link with rheumatic heart disease, which the majority had never heard of. A small amount of funding has yielded impressive results in Prof Bode-Thomas’ Small Grant project. When all the consultations, reviews and discussions were completed, final products so far include:

- An educational manual on the progression of disease from Sore Throat to ARF to Rheumatic Heart Disease for health workers to conduct patient and public education.
- Patient educational leaflets on Secondary Prophylaxis/Benzathine Penicillin (BZP) and Anticoagulation Therapy (Warfarin/INR monitoring) after valve surgery.
- Audio jingles in English, Hausa and Berom languages for public education.
- RHD patient information and advocacy leaflets in English and Hausa
- Community awareness posters in English and Hausa

Permission was granted to publish the case study extract 1 above.

**Case extract 2: RHD Action Small Grant Supports Efforts for National Roll-Out of RHD Care in the Philippines**

The team created educational materials to cover important topics for facilities to meet the nationally required certification standards to become an approved RHD provider under PhilHealth. They formally launched their programme at the Children’s Heart Foundation Grand Auditorium Philippine Heart Center in Quezon City in November 2018. From October 2018 through May 2019, using live and video conferencing, the team was able to reach over 700 doctors, nurses, other allied health professionals affiliated with school health programmes (not counting all those who attended via video link) with five presentations of their Symposium for Hospitals Accredited for FREE BPN. Dr “Jing” (Lopez-Ballelos) expressed her enthusiasm and excitement: “This has never happened in Philippine history. After this approval, we wish to eradicate RF in the country within the next 10 to 20 years.” We at RHD Action wish them well and look forward to continuing to work together, supporting and advocating for all efforts aimed at preventing and controlling RHD across the globe.

Permission was granted to publish the case study extract 2 above.

### Future opportunity and recommendations

The grant recipients were invited to share their reflection on future opportunities that could open from the Small Grant Programme and to make recommendations on how the program could be improved. Below is a selection of quotes from the recipients:

What we want to do is aim to conduct training for these community health care workers because they are at the grass root level. In fact, community are our ears and eyes and they are our contact to all our RHD patients out there in the communities.” (Respondent 12)

“The grant created opportunity for a forum where people who are interested can come together and share ideas on how their experience was and what worked and how we can apply it in our country. That will probably create a difference (Respondent 4).

recent years some of my mentees have wanted to apply and have had great ideas, but I think it has become if I remember correctly a little narrower in the scope. I forget what the actually scope was last year, but you know he had some great ideas, but it didn’t really fit with what the grant wanted that year, so I would suggest broadening the scope as future recommendation (Respondent 10).

But to improve, maybe in the more future, if the amount of money would be more, so the larger population could benefit but then generally I liked the grant because the response has been very good (Respondent 9).

## Conclusion

For RHD Action, there is large return on a modest monetary investment resulting in a very visible, viable global RHD networking platform for enthusiastic community and provider activists. The Small Grants Programme has been very effective at mobilizing grass roots community action in multiple world regions, motivating front-line health care workers and raising awareness of RHD in the community. As a direct consequence the work of the grant recipients has become more effective and efficient. While successful at the community level, engaging government stakeholders has proved challenging. If engagement of government continues to be a stated aim of the programme, further advice and support will be needed from RHD Action. This level of support for grant recipients was not built into the original programme budget.

In the absence of scaled up investment in RHD prevention and management by governments, the RHD Action Small Grants Programme is one of the only opportunities for people working in RHD in LMICs to access resources to tackle RHD.

## Additional Files

The additional files for this article can be found as follows:

10.5334/gh.996.s1 Supplementary File. Quantitative questionnaire survey.

