# Supplementary material for: The RHD Action Small Grants Programme: Small Investment, Big Return!
Source: Glob Heart. 2021 Apr 27;16(1):28. doi: 10.5334/gh.996 (PMC8086735; doi:10.5334/gh.996)
Supplement: Supplementary File. — Quantitative questionnaire survey. [file gh-16-1-996-s1.pdf]

1. Quantitative questionnaire survey

This online survey is being carried out by the Reach team based at the University of Cape Town on behalf of RHD Action. We want to evaluate the impact and effectiveness of the RHD Action Small Grants Programme. We are asking individuals whose projects were successfully funded to provide valuable feedback by completing this survey. The results of your feedback will be used to assess the impact of the Programme, adjust and improve the programme, and to potentially use as justification to request continued funding beyond its current mandate.

Instructions

The survey will start by asking you basic information about your project and then brief statements about successes and challenges you may have experienced. We also want to hear about your experience with the RHD Action Small Grants process – applying for the grant, support you may or may not have received to carry out your project, the reporting process, any media coverage provided.

It should take between 15-25 minutes to complete the survey, depending on the nature of your project. Please try to complete the questionnaire within the next two weeks.

To navigate around the survey, use the buttons at the bottom of the page. The reset button will clear responses you have entered on a single page. Responses entered on other pages will be unaffected.

We would like to follow up your responses with a personal phone interview. Please indicate at the end of the questionnaire if you are willing to be contacted and if yes, provide the best medium of communication and times for contacting you.

Don't forget to click *Submit* when you reach the end of the questionnaire.

Thanking you in advance,

The Cape Town Reach Team

1. Your name:

____

2. Organisation Name:

____

3. Project Location (City/Town/Province/Country):

____

4. Project name:

____

5. Year of RHD Action Grant:

   ☐2017 ☐2018 ☐2019

6. What was your project title?

7. ____

8. What was the general aim and activity of your project?

   ____

   (i.e., Health Worker Training, Community Awareness Raising Campaign, Support for PLWRHD, Education/Awareness Raising for School Children and Teachers, Government/MOH-Related Initiative)

9. Who were your project beneficiaries? How many beneficiaries did you reach?

People Living with RHD (PLWRD)

☐ Paediatric Patients (10 years and under) Numbers Reached: _____

☐ Adolescent Patients (11 to 18 years) Numbers Reached: _____

☐ Adult Patients (19 years and older) Numbers Reached: _____

☐ Pregnant Women & Women of Child-bearing Age Numbers Reached: _____

Schools and Communities

☐ Patients' Families and/or Community Members Numbers Reached: _____

☐ School Children, Teachers or other School Personnel Numbers Reached: _____

Health Care Providers and Public Officials

☐ Health Care Providers Numbers Reached: _____

☐ MOH/Government Officials Numbers Reached: _____

☐ **Others. Please describe who they were**.

____________________________________ Numbers Reached: _____

____________________________________ Numbers Reached: _____

If you provided training to Health Care Providers, please complete the section below.

☐ Nursing Staff Numbers Reached: _____

☐ Doctors Numbers Reached: _____

☐ Medical Students Numbers Reached: _____

☐ Nursing or Other Allied Health Students: Numbers Reached: _____

☐ Others: Please describe: ___________________ Numbers Reached: _____

10. What special skills or topics were they trained in? (TICK ALL THAT APPLY)

☐ BPG Administration

☐ Recognising and Treating Strep Throat/Pharyngitis

☐ Recognising and Treating ARF

☐ Recognising and Treating RHD

☐ Pregnancy and RHD

☐ Caring for Valve Surgery Patients (pre and/or post)

☐Anticoagulation Management

☐Others: Please describe: ___________________

11. Have you completed your project? ☐ Yes ☐ No

If yes, when: ________________ (Month/Year)

12. Did you promote your project using digital media in addition to the media coverage provided by RHD Action?

☐ Yes ☐ No

13. If yes, which of the following did you use? (TICK ALL THAT APPLY)

☐ Your organization/group's own website

☐ A new website created for the project

☐ Facebook

☐ Twitter

☐ Newsletter

☐ Posters and Flyers

☐ Local TV and Radio

☐ Others: Please specify ________________________________

14. Did you work in partnership with any other organisations/institutions/government officials in the delivery of your project?

☐Yes ☐No

If yes, please tell us the name of your partner(s) and where they are located.

____

15. How would you describe the importance of RHD Action funding to your project?

If we had not received the funding...

☐ The project would have gone ahead as planned using alternative sources of funding.

☐ The project would have been delayed whilst alternative sources of funding were sought.

☐ The scope of the project would have been reduced because of reduced funding available.

☐ The project would not have gone ahead at all.

☐ Other. Please describe: ____

THE PROCESS

16. How did you find out that this funding was available?

☐ Newsletter

☐ Email Blast

☐ Forwarded from a colleague, co-worker or supervisor

☐ Twitter

☐ FaceBook

☐ Website

☐ Other. Please specify. ____________________

17. Overall, how useful did you find the RFP and Guidelines for the application process?

Not helpful at all	1	2	3	4	5	Very Helpful

18. Overall, how did you find the timeline from application to finishing up your project (from the application to the awarding of the grant)?

Not Efficient/Appropriate	1	2	3	4	5	Very Efficient

19. Overall, how did you find the support provided by RHD Action to carry out your project?

Not Helpful/Responsive	1	2	3	4	5	Very Helpful/Responsive

20. Overall, how did you find the media coverage provided by RHD Action to carry out your project?

No/Poor Media Coverage	1	2	3	4	5	Great Media Coverage

21. Overall, how did you find the reporting process required by RHD Action?

Burdensome/Unreasonable 1 2 3 4 5 Very Reasonable/Doable

Short Answer Questions – 50-word limitation per question. These topics may be discussed in more detail in a phone interview.

22. What motivated you and/or your organization to do your project?

23. What would you describe as the main or most important outcome of your project?

24. What were the greatest successes of your project?

25. What were the most difficult challenges you faced while delivering your project?

26. Was there any unintended outcome that surprised you about your project?

27. What do you think the lasting benefits of your project will be?

28. Please provide feedback, positive or negative, on the support provided by RHD Action for your project.

29. Now that you have finished your RHD Action Small Grant project, what does your organisation plan to do next?

30. May we contact you to schedule a more in-depth discussion about your RHD Action Small Grant experience?

   ☐Yes, please ☐No, thank you.

   If yes, please provide the best contact details and medium of communication for you.

Phone number: ______________________________

Skype Name: ______________________________

What’s App: ______________________________

Viber: ______________________________

Best Email: ______________________________

Other Options: ______________________________

Best times and day of week for call: ______________________________

Your Time Zone: ______________________________

Thank you for your feedback. Please click submit below. Thank you.
